# Development, delivery, and evaluation of the Texas Epidemic Public Health Institute pilot infection control lecture series

**DOI:** 10.3389/fpubh.2025.1534560

**Published:** 2025-03-12

**Authors:** Kayla E. Ruch, Anabel Rodriguez, Janelle Rios, Luis Ostrosky-Zeichner, Eric L. Brown

**Affiliations:** ^1^Texas Epidemic Public Health Institute Infection Prevention and Control Program Manager, Department of Epidemiology, The University of Texas Health Science Center at Houston, School of Public Health, Houston, TX, United States; ^2^Department of Environmental and Occupational Health, School of Public Health, Texas A&M University, College Station, TX, United States; ^3^Department of Environmental and Occupational Health Sciences, Texas Epidemic Public Health Institute Houston, The University of Texas Health Science Center at Houston, Houston, TX, United States; ^4^Department of Internal Medicine, McGovern Medical School, University of Texas Health Science Center at Houston, Houston, TX, United States; ^5^Department of Epidemiology, School of Public Health, University of Texas Health Science Center at Houston, Houston, TX, United States

**Keywords:** IPC education, infection preventionist, training, healthcare-associated infection, occupational health and safety

## Abstract

**Introduction:**

The Texas Epidemic Public Health Institute (TEPHI) aims to keep Texans healthy and the economy strong by preparing for the next infectious disease outbreak. TEPHI’s Small Rural Healthcare Preparedness core developed, delivered, and evaluated a pilot infection prevention and control webinar series called *Infection Control* for rural-serving health professionals and organizations based on infection prevention and control field best practices.

**Methods:**

Data from the first year of the Infection Control series was collected through attendee registration forms, attendance records, knowledge, and post-lecture evaluation surveys using Qualtrics. The data were analyzed using Qualtrics software. Lectures were free and open to the public across disciplines. The material was promoted through public health channels with promotional flyers.

**Results:**

1,105 individuals attended or viewed the Infection Control series. Despite a generally low response rate to evaluation surveys, feedback was consistently positive. Participants noted a “high likelihood of future TEPHI infection prevention and control lecture attendance.” The feedback informed improvements for the second year of the series.

**Conclusion:**

Attendees of the *Infection Control* series gained a deeper understanding of relevant policies, procedures, and practices. By providing essential, accessible education on infection prevention and control at no cost, healthcare systems, administrators, and providers in rural healthcare systems across Texas have acquired the necessary knowledge to establish and maintain a safe environment for patients and staff in healthcare settings.

## Introduction

The Texas Epidemic Public Health Institute (TEPHI) is a state agency of higher education headquartered at The University of Texas Health Science Center at Houston, Texas (UTHealth Houston) in the United States. TEPHI’s mission is to keep Texans healthy and the economy strong by strengthening the capacity and resiliency of Texas communities to respond to future infectious disease outbreaks. TEPHI works to enhance and support a robust, well-trained public health workforce, prepare Texans for public health threats related to contagious diseases, and promote a strong economy resilient to infectious diseases within the state. Texas is currently the second most populous state in the United States, with a population of over 30 million residents as of 2024 and comprised of 254 counties (1). The overarching goals of the TEPHI Infection Prevention and Control Webinar lecture series are to (2) provide small rural hospitals with infection prevention education and readiness training and (3) provide rural communities with accurate and scientifically sound resources they can use to prevent and mitigate the impact of infectious disease and avoid overwhelming small rural hospital capacities (2).

Senate Bill (S. B) 1780 was passed in May 2021 with bipartisan support by the 87th Texas Legislature, establishing TEPHI to prepare Texans to mitigate the impact of infectious diseases (1). Governor Abbott signed the bill into law on June 16, 2021. The University of Texas System Board of Regents approved the establishment of TEPHI on August 18, 2021 (2). Senate Bill (S. B) 8, passed by the 87th Legislature on October 19, 2021, during its third called session, appropriated funds from the Coronavirus State Fiscal Recovery Fund to implement and maintain TEPHI over the 2022–2023 biennium (2). In response to the COVID-19 pandemic, Texas introduced SB 1780 to strengthen public health preparedness for future infectious disease outbreaks (2).

TEPHI was created to support a robust public health workforce, invest in statewide public health readiness related to infectious diseases, and promote evidence-based protocols and communication – all with a distinct focus on preparedness (2). The legislative charge was to help Texas better prepare for the next pandemic while assisting with efforts to transition out of the COVID-19 pandemic. To do this, TEPHI initially developed three core areas of readiness, training, and communications, with each project core grounded in collaboration (2). More recently, the readiness core was divided into two distinct areas: early detection initiatives and the public health reserve network. The Small Rural Healthcare Systems working group is part of the public health reserve network.

The Infection Prevention and Control Webinar series material is designed for infection preventionists, public health professionals, and healthcare workers responsible for infection prevention and control in healthcare facilities. Globally, infection prevention and control programs training and programs vary from dependently on many factors such as resource availability such a clean water, healthcare working staffing, medical equipment, medical service lines within the facilities, and environmental factors. The Global Infection Prevention Control (GIPC) Network aims to enhance coordination and collaboration in infection prevention and control (IPC) at local, national, and international levels (3). It supports WHO and Member States in strengthening IPC systems and programs, outbreak prevention and control, capacity building, and surveillance (reference). Many organizations participate in the (GIPC) such as the Asia Pacific Society of Infection Control (APSIC), Association for Professionals in Infection Control and Epidemiology (APIC), European Committee on Infection Control (EUCIC) organization, Infection Control Africa Network (ICAN) and others (3). There is no current standardized training for infection prevention and control practices in the United States in healthcare settings. Infection prevention and control (IPC) measures vary across states due to local regulations, healthcare infrastructure, and public health priorities (4, 5). While national guidelines, like those from the Centers for Disease Control and Prevention (CDC), provide a foundational framework, states often adapt these recommendations to address specific regional needs and circumstances (4). Due to Texas’s needs, the Small Rural Healthcare Preparedness working group developed a pilot educational lecture series to provide infection prevention and control information to help improve Texas preparedness levels for the next infectious pandemic with the Infection Prevention and Control webinar lecture series (2).

This paper will review the pilot year one Infection Control series by analyzing module registration information, attendance numbers, YouTube views, and post-survey evaluations. The goal is to identify strengths, opportunities for improvement, and recommendations for year two of the TEPHI Infection Prevention and Control Pilot lecture series.

## Materials and methods

The lecture material was based on the eight core components of Certification in Infection Control and Epidemiology (CIC), developed by the Certification Board of Infection Control and Epidemiology (CBIC), Inc. (6, 7). The CIC® examination is an industry-standard metric measuring the core knowledge, skills, and abilities of infection preventionists (6, 7). Although optional, it is regarded as the benchmark for best practices in the field. The eight core components are identification of infection disease processes, surveillance and epidemiology investigations, preventing/controlling the transmission of infectious agents, employee/occupational health, management and communication, education and research, the environment of care and cleaning, sterilization, disinfection, and asepsis (6, 7). The TEPHI Infection Prevention and Control lecture series covers the material from eight core components in the five-lecture webinar lecture series.

This study involved pilot data analysis from the first year of the Infection Control series. Data were sourced from TEPHI webinar lecture registration forms and attendance records, which are maintained on the WebEx Webinar Platform® (8). Registration statistics, including registration, attendance, and YouTube viewer data, were collected on Modules 101–105. Additionally, demographic data from Modules 102–105 registrants were collected, requiring participants to provide their names, email addresses, attendance type (in-person or virtual), credentials, organizational affiliations, job titles, and years of experience in infection prevention and control. A comprehensive submission of information was mandated by the registration algorithm. Descriptive statistics were subsequently computed using Qualtrics® (9). This pilot project was funded from *S. B. 1780, 87th Legislature, 2021 Reg. Session.* The authors reported no potential conflict of interest.

The infection prevention and control lecture material was based on the eight core components. Resource material was pulled from multiple sources such as The Association for Professionals in Infection Control and Epidemiology Text (APIC Text), the Centers for Disease Control and Prevention (CDC), the Occupational Safety and Health Administration (OSHA), and The Joint Commission Regulations (TJC) to address fundamental components of infection prevention and control training. The pilot project developed five 60 minutes lectures to cover multiple components. All Modules were presented in-person and online via WebEx® (8). All modules were at no cost and recorded on the TEPHI YouTube channel. Table 1 provides an overview of each module. The presentation slide deck and link to the lecture were provided to attendees for future reference. Institutional Review Board (IRB) approval was not required for publicly available education series since they do not involve research with human subjects nor involve the collection of protected health information or intervention research initiatives.

**Table 1:** Infection control webinar module overview.

Module number and title	Overview	Learning objectives	Additional material
Module 101: foundations of infection prevention and control	Provides an overview of foundational infection prevention and control components.	- Hand hygiene practices - PPE use (including donning and doffing) - Transmission chains - Texas notifiable public health conditions - Effective infection prevention and control in healthcare settings	Material presented from CDC and Texas specific notifiable conditions (16)
Module 102: preparedness	Provides an overview of infection prevention risk assessments, resource management, and infectious waste management in healthcare facilities.	- Explain the elements of infection prevention and control risk assessment - Effective resource management practices - Describe essential waste management best practices	University of Texas Medical Branch (UTMB), SPECTRE program collaboration on, lessons learned in infectious disease from field experience (17)
Module 103: environment of care	Provides an overview of environmental disinfection, the Joint Commission environment of care regulations, and construction and renovation practices using infection control risk assessment for healthcare facilities.	- Describe environmental cleaning, disinfection, and sterilization practices - Identify safe environment of care practices - Construction and renovation practices in healthcare settings	Joint Commission regulations and the Construction Infection Control Risk Assessment (ICRA) discussed in detail (18).
Module 104: infection prevention and control programs	Provides an overview of infection prevention and control programs, conducting surveillance and epidemiology investigations, and management and communication practices in healthcare facilities.	- Describe infection prevention and control programs in healthcare settings - Describe epidemiological investigations in healthcare settings - Discuss effective communication and management practices of infection prevention and control programs	Different program components such as surveillance practices, isolation compliance, high-level disinfection practices, sterilization practices, and epidemiological outbreak investigation processes discussed (19)
Module 105: National Healthcare Safety Network (NHSN)	Provides a brief introduction to the National Healthcare Safety Network, Central Line-Associated Bloodstream Infections, Catheter-Associated Urinary Tract Infections, and Surgical Site Infections using NHSN 2023 manual.	- Explain the National Healthcare Safety Network (NHSN) - Discuss Central Line-Associated Bloodstream Infections (CLABSIs), Catheter-Associated Urinary Tract Infections (CAUTIs), and Surgical Site Infections (SSIs).	A brief description of each NHSN Healthcare-Associated Infection (HAI) criterion was given (20)

After each module, participants were asked to complete an anonymous post-evaluation survey on the material presented to reduce respondent bias. To enhance the response rate for Modules 104-105, TEPHI merchandise incentives were introduced. Participants who completed the survey were eligible to enter a lottery to win TEPHI merchandise. Furthermore, TEPHI merchandise was mailed to one selected participant from each of Modules 104 and 105. The post-evaluation survey was developed via email link and QR code at the end of each module, and data was stored in Qualtrics® (9). Descriptive analyses were generated using Qualtrics software, Version 2020 of Qualtrics (9). The survey was comprised of 12 closed-ended questions and two open-ended questions. Questions ranged from rating the lecture core based on the Likert scale response to rating one through five, with five being the highest. Closed-end questions scoring 1–5 with 5 = strongly agree, 4 = agree, 3 = neutral, 2 = disagree, and 1 = strongly disagree responses were asked regarding content clarity, usefulness, and impact. Measures of Central Tendency were calculated. The post-evaluation survey was emailed to the attendees, and attendees could scan the QR code from the presentation slides. Statistics averages were calculated on the post-evaluation survey responses. Participants’ responses to the open-ended question (i.e., *What additional topics would individuals like to see presented at future seminars?*) were used to develop year two of infection prevention and control content. Modules 101–105 were approved for 1.0 continuing education credit hours from the National Board of Public Health Examiners (NBPHE) for individuals with a public health certification (CPH) (10). Modules 103 and 105 were approved for 1.0 continue education credit from the Certification Board of Infection Control (CBIC) for individuals with a certification in infection control and epidemiology (CIC®) (11). Modules 101–105 were approved for continuing education credit from the Certification Board of Infection Control (CBIC) for individuals with a certification associate-infection control and epidemiology (a-IPC^©^) (12).

## Results

Module 101 had the highest registrations, attendances, and YouTube views, with 295, 180, and 220, respectively (Table 2). Module 105 had the lowest engagement, with 132 and 57 individuals. Module 102 has the second-lowest number of registrants (182) and attendance (93); however, it achieved the second-highest number of YouTube views. Module 103 had the second-highest registrants (277) and second-highest attendance level (138). Cumulatively, the total viewership for the series, encompassing live attendance and YouTube views as of December 31, 2023, was 1,105. The comprehensive total for the year, combining registrants, attendees, and YouTube viewers, reached 2,275 individuals.

**Table 2:** Modular registration, attendance, and YouTube views.

Module	Date presented	Registrants	Attendees	YouTube views (12/31/23)	Total view (attendee + YouTube)
Module 101	March 22, 2023	295	180	220	400
Module 102	May 17, 2023	182	93	122	215
Module 103	July 19, 2023	277	138	67	205
Module 104	September 20, 2023	284	116	64	180
Module 105	November 1, 2023	132	57	48	105
Year 1 total		1,170	584	521	1,105

Registration attendance demographic information varied throughout the lecture series (Table 3). The average age of registered participants was consistently in the 30–39 range throughout the lecture series. For module 104, two individuals reported an age of <20, considered an outlier. The smallest age distribution group was the 60+ category through the series. Throughout the series, consistently more females registered for the lecture series, with a total female registration of 672 compared to 182 males, a 3.7: 1 ratio in registration.

**Table 3:** Infection control series attendee demographic and occupational characteristics.

Characteristics	Module 2 (*n* = 182)	Module 3** (*n* = 277)	Module 4 (*n* = 284)	Module 5 (*n* = 132)
	*n* (%)			
Age				
20–29	42 (23.1)	48 (17.3%)	67 (24%)	26 (19.7%)
30–39	52 (28.6%)	74 (26.7%)	87 (31%)	50 (37.9%)
40–49	38 (20.9%)	63 (22.7%)	61 (21%)	25 (18.9%)
50–59	31 (17.0%)	51 (18.4%)	44 (15%)	19 (14.4%)
60+	13 (7.1%)	30 (10.8%)	11 (4%)	5 (3.8%)
PNA*	6 (3.3%)	11 (4.0%)	12 (4%)	7 (5.3%)
Gender				
Female	145 (79.7%)	212 (76.5%)	214 (74%)	101 (76.5%)
Male	32 (17.6%)	57 (20.6%)	64 (24%)	29 (22%)
PNA*	5 (2.7%)	8 (2.9%)	6 (2%)	2 (1.5%)
Race				
American Indian or Alaskan Native	3 (1.6%)	4 (1.4%)	5 (1.5%)	2 (1.5%)
Asian	23 (12.6%)	40 (14.4%)	55 (19.4%)	30 (22.7%)
Black or African American	24 (13.2%)	43 (15.5%)	38 (13.4%)	22 (16.7%)
White	116 (63.7%)	156 (56.3%)	136 (48%)	55 (41.7%)
2 or More Races	16 (8.8%)	8 (3%)	7 (2.5%)	6 (4.5%)
PNA*		26 (9.4%)	43 (15.2%)	17 (12.9%)
Ethnicity				
Hispanic or Latino	51 (28.0%)	68 (24.5%)	57 (20%)	35 (26.5%)
Not Hispanic or Latino	115 (63.2%)	155 (56.0%)	171 (60%)	75 (56.8%)
Other	7 (3.8%)	36 (13.0%)	56 (20%)	22 (16.7%)
PNA*	9 (5.0%)	18 (6.5%)		
Years in current position				
0–2 years	105 (57.7%)	162 (58.4%)	155 (54.6%)	84 (63.6%)
3–5 years	44 (24.2%)	77 (27.8%)	65 (22.9%)	32 (24.2%)
6–10 years	20 (11%)	19 (6.9%)	28 (9.8%)	8 (6.1%)
10+ years	13 (7.1%)	19 (6.9%)	36 (12.7%)	8 (6.1%)
Years in infection prevention				
0–2 years	74 (40.7%)	90 (32.5%)	124 (43.7%)	59 (44.7%)
3–5 years	53 (29.1%)	81 (29.3%)	75 (26.4%)	46 (34.8%)
6–10 years	27 (14.8%)	48 (17.3%)	38 (13.4%)	15 (11.4%)
10+ years	28 (15.4%)	58 (20.9%)	47 (16.5%)	12 (9.1%)
Military service				
Yes	Not collected	19 (6.9%)	18 (6%)	9 (7%)
No		239 (86.2%)	227 (80%)	106 (80%)
PNA*		19 (6.9%)	39 (14%)	17 (13%)

Module 1 registration data not collected. Military Status for Module 1 data not collected. *PNA, preferred not to answer. **2 individuals reported < 20 years for Module 103.

A small percentage of individuals (<2.9 or smaller) in each module did not report gender. The racial composition was primarily White, followed by Asian and Black or African American participants. A majority identified as Not Hispanic or Latino, with an average of 24.6% reporting Hispanic or Latino ethnicity. Percentages of years in current position and years in infection prevention followed the same pattern, with most registrants in 0–2 years of practice. For Modules 101–103, positions showed a consistent pattern of individuals reporting most frequently being in 0–2 years, 3–5 years, 10+ years, and 6–10 years. However, in Module 104, individuals reported 0–2 years most frequently, with 10+ years being the least reported. The variation in reported numbers shows that individuals have been in infection prevention and control for different amounts compared to their current position. Lastly, military service was reported for Modules 102–105, with service members registering 6–7% and most non-service members registering for modules. Most participants lacked certification in infection prevention and control (CIC®), with only seven individuals reporting that they had obtained it. Demographic analysis indicated that the majority of professionals in the field were female, White, and not Hispanic or Latino. There were fewer male participants and fewer individuals of Hispanic or Latino ethnicity or military background. Additionally, most professionals reported having between 0 and 5 years of experience in infection prevention and control.

Table 4 describes responses from individuals who completed the post-module survey that provided feedback on the 60 minutes infection prevention lecture. The series’ response rate improved, with the peak response at Module 104. Virtual attendance dominated all modules, with Modules 103 and 105 exclusively virtual. Individuals reported that the perceived benefit and clarity of the material were consistently rated high across all modules. Of the individuals who responded, 96% of participants across all modules indicated they would implement the knowledge gained in their organization. The high ratings for the material’s benefit, clarity, and intent to implement the knowledge suggest that the seminars are well-received overall. Individuals requested the following topics be presented on future TEPHI modules: Surveillance, Hazard, Lyme Disease, Back safety/ergonomics, Foodborne Illness related topics, quality improvement projects focused on infection control, Case studies, Emerging Diseases, Sanitation and hygiene, hemodialysis, Data Analysis, GIS, gap analysis, epidemiology (3 responses), surveillance (3 responses), sterile processors, outbreak, Occ. health, career advancement, NHSN incision definition and information.

**Table 4:** Modular post-survey evaluations.

Evaluation questions	Module 1 (*n* = 180)	Module 2 (*n* = 93)	Module 3 (*n* = 183)	Module 4 (*n* = 116)	Module 5 (*n* = 57)
	*n* (%)				
Response rate	9 (5%)	4 (4.30%)	12 (8.7%)	36 (31%)	14 (25%)
Attendance					
In-person	2 (22%)	1 (25%)	0 (0%)	1 (3%)	0 (0%)
Virtual	7 (78%)	3 (75%)	12 (100%)	35 (97%)	14 (100%)
Module Rates					
The material presented was beneficial? (Rate 1–5)	5	5	4.55	4.89	4.95
The amount of content covered was appropriate? (Rate 1–5)	5	4.75	4.27	4.89	4.9
Was the material being easy to understand? (Rate 1–5)	4.78	4.75	4.45	4.92	4.71
Was the material clear and concise? (Rate 1–5)	4.89	4.75	4.36	4.89	4.86
Will you implement the knowledge gained from this seminar at your organization?					
Yes	6 (67%)	4 (100%)	9 (75%)	34 (94%)	12 (86%)
No	0 (0%)		1 (8.3%)	1 (3%)	1 (7%)
Missing**	2 (22%)		2 (16.7%)	1 (3%)	1 (7%)
PNA*	1 (11%)				
Do you plan to attend future infection prevention seminar series modules?					
Yes	7 (78%)	4 (100%)	10 (83%)	35 (97%)	13 (93%)
No	0		0	1 (3%)	1 (7%)
Missing**	2 (22%)		2 (17%)		

*PNA, preferred not to answer. **Missing due to individuals not providing a response.

## Discussion

The Texas Epidemic Public Health Institute’s Pilot Infection Prevention and Control lecture series was designed to enhance infection prevention education and readiness for small rural hospitals and to provide vital local resources to rural communities heavily impacted by the pandemic (2). This initiative aimed to prevent future disease spread and alleviate the strain on rural hospital capacities by providing Texas healthcare systems with free, readily available infection prevention and control education (2). The series attracted over a thousand participants during the first nine months, with many providing positive feedback and expressing high interest in attending future TEPHI IPC webinar lectures. Participants frequently reported high satisfaction with the content and the knowledge they acquired, marking this a vital indicator of the series’ success. This positive (strongly agree) response underscored the series’ role in meeting and exceeding educational expectations and establishing a significant benchmark in overall participant satisfaction.

The majority of participants are early to mid-career professionals, predominantly aged 30–39, with 0–2 years of experience. This suggests that the training programs effectively appeal to individuals in the early stages of their careers but may need to adapt to attract more senior professionals. Future series could be differentiated by proficiency levels, offering tailored content for novices, mid-career professionals, and senior infection preventionists. Additionally, the majority of participants identified as White, non-Hispanic females, highlighting the need for targeted outreach to engage and improve representation of underrepresented racial and ethnic groups.

The first year showed a clear preference from individuals to attend modules virtually. The survey response rates did vary throughout the modules; however, when an incentive measure was implemented, survey response rates increased overall. While the ratings for “material beneficial” and “content appropriateness” are consistently high, there is a more notable fluctuation in the “ease to understand” and “clear and concise” categories, particularly with Module 103, which scored lower than the others. However, even the lowest scores are still relatively high, indicating generally positive (strongly agree) feedback across all modules. The variation in responses was not drastic, which suggested that while there were differences in the reception of each module, the overall quality was maintained at a level that participants found satisfactory. Most participants indicated a persistent level of quality and clarity on the material and gained knowledge throughout the series. Lastly, individuals’ intention to attend future modules was high. These deeper insights provide the seminar organizers with actionable feedback. The consistently high scores suggest that the seminar series meets its content quality and relevance goals. Lastly, the variations in the response rate and the slight dips in certain areas highlight opportunities for targeted improvements, such as enhancing engagement strategies, modifying content to maintain high standards, and subject-specific topic requests.

### Future program progression

Based on participant numbers and feedback from the initial lecture series, it has been decided to extend the series into its second year, with several modifications derived from insights gathered during the first year. A standardized process for registration and post-lecture surveys will be implemented at the start of year two, which will streamline data collection and reduce instances of missing data. In response to changes in societal preferences influenced by the COVID-19 pandemic and considering the high rate of virtual attendance in the first year, future modules will be offered exclusively online. This adaptation not only meets the current preferences of our audience but also extends our reach to those who may not be able to attend in person.

To further increase participant engagement, the format of the lectures will be adjusted for year two. The 60 minutes lecture will be reduced into a more focused 45 minutes presentation with an interactive 10 multiple-choice question knowledge activity based on participant feedback to increase engagement and allow the program to establish a metric to determine the effectiveness of material presented to the audience. The knowledge activity aims to foster a deeper understanding and encourage discussion among attendees. After the interactive session, the floor will be opened for questions, allowing direct interaction between the participants and the presenter. Feedback about the interactive knowledge activity will be collected from the lecture post-event survey to gage its effectiveness. This will help determine if these adjustments meet our audience’s educational needs and expectations.

Lastly, feedback provided by participants on the topics covered has been vital. This input has directly influenced the development of the lecture topics for year two. The organizers have planned 10 monthly modules from February to November 2024, each focusing on a topic selected based on participant suggestions. Below, the planned module topics are outlined to provide participants with a clear roadmap for the upcoming series.

1. Module 201: epidemiology
2. Module 202: occupational epidemiology and prevention
3. Module 203: surveillance
4. Module 204: data handling
5. Module 205: hemodialysis
6. Module 206: emerging infectious diseases
7. Module 207: outbreak
8. Module 208: contact tracing
9. Module 209: infection prevention and control collaboration
10. Module 210: quality improvement

### Study limitations

The pilot study encountered several limitations. Firstly, neither registration nor post-evaluation data were collected in a standardized manner prior to the commencement of the lecture series. As the project progressed, we developed standardized procedures for data collection and post-survey evaluations. These measures should have been established before the pilot project began. This limitation is addressed in year two by creating all registration and post-survey evaluation collection questions prior to the series presentation to eliminate missing data due to failure to collect. Next, there was an overall low response to the post-survey evaluation. Additionally, a knowledge assessment was not conducted in each module to gage learning; however, year 2 of this program has adjusted for this. Adding an incentive measure increased the response rate; however, this could have introduced bias because individuals only responded because they wanted to obtain TEPHI merchandize.

Lastly, response bias should be considered because individuals felt obligated to respond positively (strongly agree) to the feedback. This could be due to feeling obligated to provide positive (strongly agree) feedback to be selected to win TEPHI merchandize. All data collected was reported by the individual, thus allowing for self-reporting bias. The individual’s rating measures could be seen as subjective and could be influenced by many factors, such as familiarity with the topic and desire to learn about the topic.

The pilot study also had various strengths. Unlike other allied health concentrations, infection preventionists have no standardized education track, such as a traineeship or degree concentration program (13, 14). First, it introduced a pioneering approach designed to address the inconsistencies in infection prevention and control training due to information obtained primarily from the train-the-trainer model at the facility level, thus needing standardized infection prevention and control training programs. This model addresses the unique needs, objectives, and standards of the healthcare facility’s Infection Prevention (IP) department (13, 14). Through one-on-one mentorship, a senior IP professional conveys practical knowledge, institutional protocols, and infection prevention competencies tailored to the facility’s specific patient population and service lines (13, 14). However, the effectiveness of this model may be influenced by the mentor’s skill set, potentially impacting the precision and accuracy of the knowledge imparted. As the trends of professionals taking the CIC exam have increased over time, more education needs to be created and made freely accessible for individuals to prepare for certification (15) adequately. Infection prevention professionals have a wide range of expanding roles, highlighting the need for standardized and accessible training (14). Professional organizations such as APIC offer preparation materials at a financial cost; more comprehensive resources and freely available materials are needed to address variations in objective areas identified in this study (15).

The project offered additional educational resources and lecture materials free of charge, targeting professionals in healthcare settings where such resources are typically neither free nor publicly accessible. Secondly, it provided a detailed overview of the field, highlighting potential challenges professionals might face. Thirdly, the study responded directly to community needs by focusing on topics in infection prevention and control that are most desired, as determined by data-driven feedback from participants.

## Conclusion

TEPHI Infection Prevention and Control Pilot webinar series aimed to improve educational intervention by providing free and easily accessible educational material to a broad range of infection prevention and control professionals. The pilot’s objective was to identify strengths and weaknesses in program development, ascertain the needs of infection prevention and control professionals, enhance interventions and materials, and mitigate the program’s limitations. TEPHI’s first-year infection prevention and control lecture has equipped over a thousand participants with a deeper understanding of relevant policies, procedures, components, and practices related to Texas infection prevention practices. By offering essential, accessible education on infection prevention and control to the public, healthcare systems and clinicians have gained the knowledge necessary to establish and sustain a safe environment for patients and staff in healthcare settings. Ultimately, this initiative will enhance the care provided in Texas during future infectious disease outbreaks.

## Data Availability

The raw data supporting the conclusions of this article will be made available by the authors, without undue reservation.
